# Penile urethral anastomosis to the vesico‐urethral junction, after transabdominal wall passage of the penis, as treatment for intrapelvic stent‐related urethral obstruction in a dog

**DOI:** 10.1111/vsu.14249

**Published:** 2025-03-25

**Authors:** Armando Foglia, Veronica Cola, Sara Del Magno, Francesco Dondi, Roberta Troia, Stefano Zanardi, Filippo Cinti, Luciano Pisoni

**Affiliations:** ^1^ Department of Veterinary Medical Sciences University of Bologna Bologna Italy; ^2^ Small Animal Hospital, Vetsuisse Faculty Universitat Zurich Zürich Switzerland; ^3^ Clinica Veterinaria San Marco Veggiano Italy

## Abstract

**Objective:**

The objective of the present study was to report the outcome of a novel technique of urethral intra‐abdominal anastomosis after transabdominal wall passage of the penis in a dog with stent‐related urethral obstruction.

**Study design:**

Case report.

**Animal:**

A seven‐year‐old neutered male Cocker Spaniel.

**Methods:**

The dog was evaluated for urinary retention and overflow incontinence of approximately 1‐year duration. The dog had a urethral self‐expanding metallic stent placed 6 years prior as treatment for pelvic urethral stricture, secondary to severe pelvic trauma. Stent fracture and stent‐related tissue hyperplasia were diagnosed leading to intrapelvic urethral obstruction and concomitant atonic bladder complicated by cystolithiasis and urinary tract infection. An intra‐abdominal urethral anastomosis was performed to restore urethral patency, after passing the penis through the abdominal wall, into the inguinal area; the surgery was successful in bypassing the urethral obstruction.

**Results:**

No contrast leakage was noted on positive contrast cystourethrography 10 days postoperatively. The urinary bladder was easily emptied by manual expression and bethanechol was started. At 6‐months follow‐up, the urinary bladder remained atonic but was easily emptied by manual expression, with mild urinary incontinence remaining. No signs of recurrent urinary tract infections were noted. Nine months after surgery the dog was euthanized for reasons unrelated to the surgery.

**Conclusion:**

The transabdominal wall urethral anastomosis, after penile abdominal tunnelization resulted in bypassing the urethral obstruction in this dog, restoring urethral patency. The technique reported could be a viable surgical option for restoring urethral patency in dogs with severe pelvic urethral damage or obstructive lesions.

## 1 INTRODUCTION

Urethral obstruction in dogs can be caused by both benign and malignant disease.^1^

Treatment options depend on the causes of obstruction and include urethrostomy proximal to the affected site, resection and anastomosis, stent placement, permanent cystostomy tube, and urethral replacement.^1^, ^2^ The site and degree of the obstruction and the length of the remaining urethra dictate the choice of surgical technique. Intrapelvic urethral occlusion requires either a prepubic or a transpelvic urethrostomy, which are often associated with a higher complication rate than scrotal urethrostomy, especially stenosis of the external stoma, recurrent urinary tract infections (UTIs), skin scalding, urine dribbling, and urinary incontinence.^1^, ^3^ Instead, surgical techniques bypassing the obstructed region and preserving the distal urethra could reduce the rate of these complications.^1^

Urethral resection and anastomosis can re‐establish the normal anatomy of the urinary tract; however, the ability to perform this procedure is often limited by the availability of urethral tissue at the site of the obstruction and it may be more challenging to perform if the affected site is within the pelvic canal.^1^ Several modifications of traditional techniques have been described including various preputial urethrostomies, involving or not a partial or complete penile amputation^3^; end to end urethral anastomosis after inguinal tunnelization of the urethra^4^ and urethral anastomosis between the penile bulb and the neck of the bladder.^5^

Extra‐pelvic urethral anastomosis, as previously described, can be indicated when an extensive portion of the ischiatic urethra need requires excision. The procedure requires externalization of the pelvic urethra through an anatomic passage, such as the inguinal canal, before completing the urethral anastomosis to the penile urethra, assuming that the length of the remaining urethra is adequate.^4^ If the proximal urethra is too short to prevent its mobilization, a minimally invasive technique, such as urethral stenting, could be a viable option to permanently restore urethral patency. However, stents which lack a covering become easily incorporated into the urethral wall with the development of urothelial hyperplasia and in‐growth through the stent interstices potentially leading to secondary urethral obstruction.^6^ In humans, the management of this complication could be challenging and surgery with urethroplasty or definitive urethrostomy, is required in 33%–45% of cases.^6^

In the present study, the authors report the surgical technique and outcome of an intra‐abdominal urethral anastomosis after penile transposition, to manage a stent‐related complication leading to urethral obstruction in a dog, in which the healthy proximal urethra was too short to allow the use of the surgical techniques already published in the veterinary literature. To the best of the authors' knowledge, the use of surgical intervention consisting of penile urethral anastomosis to the vesicourethral junction, after transabdominal wall passage of the penis, has not been previously described for treating this unusual complication in dogs.

## 2 MATERIALS AND METHODS

A 7‐year‐old neutered male Cocker Spaniel was presented to the Veterinary Hospital of the University of Bologna with a 1‐year history of dysuria and urinary retention. Six years before consultation, the patient had severe pelvic trauma which included multiple pelvic fractures and intrapelvic injuries requiring a total prostatectomy and pubectomy. Three months after surgery, an uncovered urethral self‐expanding metallic stent was placed at the level of the previous prostatectomy as treatment for the development of a urethral stricture. Subsequent to the urethral stent application, the dog had severe urinary incontinence (1 of 10 based on the continence score proposed by Rose et al.)^7^ and recurring UTIs and was repeatedly treated with antimicrobials. The dog had also been diagnosed with idiopathic epilepsy and was currently being treated with phenobarbital (7.5 mg/kg every 8 h) and potassium bromide (16 mg/kg every 12 h).

On physical examination, an overdistended urinary bladder was detected on abdominal palpation and continuous urine dripping was noted. Urine staining of the pelvic limbs and dermatitis over the ventral aspect of the abdomen with patchy erythema, consistent with urine scalding, were also present. Digital rectal examination revealed irregular thickening of the periurethral tissue at the level of the urethral stent. Manual expression of the urinary bladder was unsuccessful. Neurological examination was unremarkable.

The results of the complete blood count and the serum chemistry were within reference intervals. Urinalysis obtained via ultrasound‐guided cystocentesis revealed urine pH of 7 and urine specific gravity of 1.021. Microscopic urine sediment examination revealed too numerous to count red blood cells, 8–10 white blood cells/high power field and bacteriuria. Urine culture was performed and *Staphylococcus pseudointermedius*, susceptible to marbofloxacin, was isolated.

**FIGURE 1:**
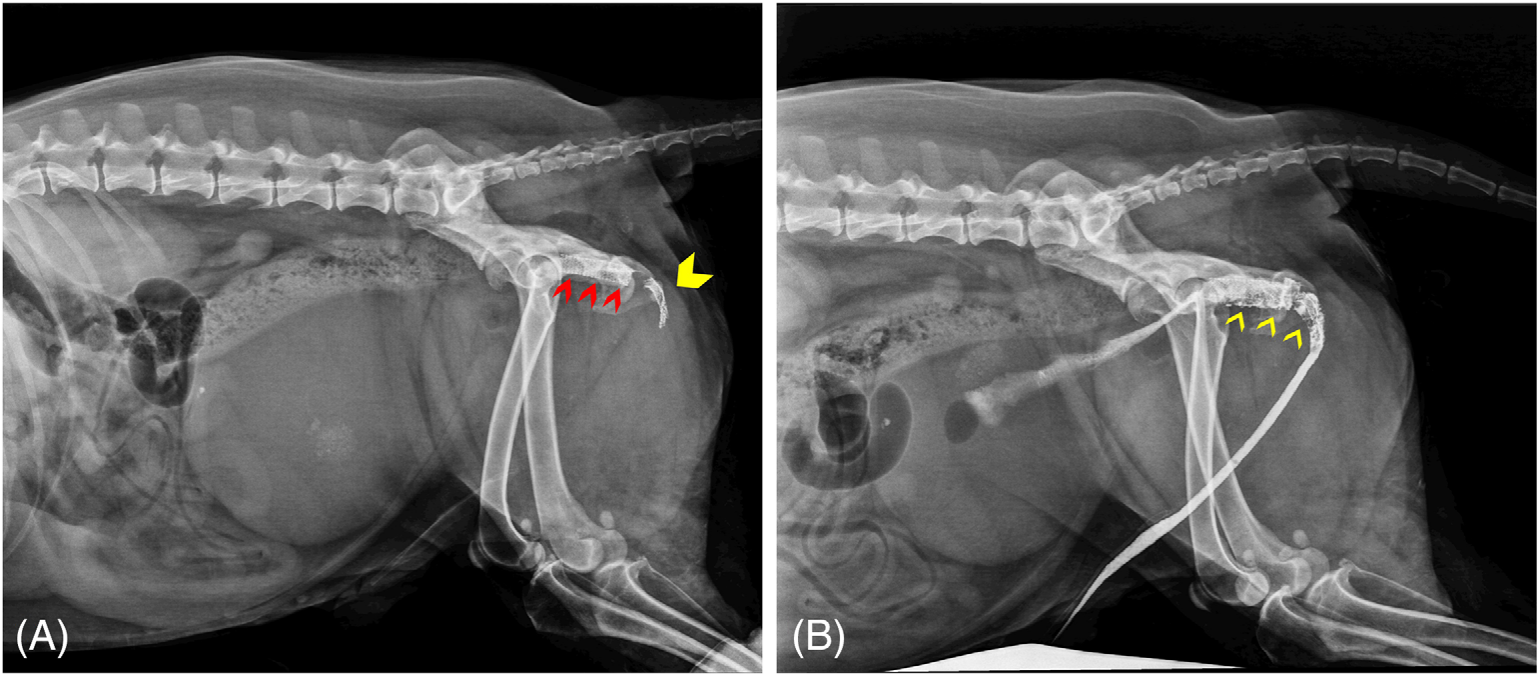
Abdominal radiographs. (A) Right lateral radiographic view. Note the urethral self‐expanding metallic stent (red arrowheads) located along the pelvic urethra and the fragment of the stent (yellow arrowhead) displaced at the level of the urethral ischiatic curvature. The urinary bladder appears to be overdistended with numerous small intraluminal uroliths. (B) Retrograde contrast cystourethrography. Note the multiple filling defects presumably related to tissue hyperplasia (yellow arrowheads). The pubis is not evident due the previous pubectomy.

Plain abdominal radiographs showed numerous, millimetric, and faintly radiopaque uroliths within an overdistended urinary bladder; no radiopaque urethral calculi were identified. The self‐expanding metallic stent, placed along the pelvic urethra, appeared to be fractured with a fragment displaced distally at the level of the ischial arch (Figure 1A). Abdominal ultrasonography revealed only the overdistended urinary bladder with floating intraluminal debris and multiple uroliths.

Retrograde urethrocystography was performed under fluoroscopic guidance. A 5 French (F) urinary catheter was inserted into the urethra; resistance was encountered at the region of the ischial arch. An approximate 50:50 mixture of iodinated contrast medium (Xenetix 300. Guerbet BP 57400‐95943 Roissy CdG Cedex‐France) and sterile saline was infused; an abrupt decrease in contrast flow within the caudal penile and intrapelvic urethra was evident at the level of the urethral stent. Subsequently, the contrast medium was seen entering the neck of the overdistended bladder (Figure 1B). An attempt to pass a hydrophilic 0.035″ guide wire (0.035‐inch flexible nitinol guidewire, hydrophilic coated, Teleflex Medical, Wayne, Pennsylvania) through the urethra retrogradely was made, with the intention of catheterizing the urinary bladder; however, it failed. Percutaneous decompressive cystocentesis, using a 22‐gauge needle, was then carried out with ultrasonographic guidance. Urethroscopy, executed with a ureteroscope (Karl Storz Flex‐ X2S Karl Storz Endoskope, Tuttlingen, Germany) revealed multiple large, pedunculated soft tissue masses at the end of the urethral stent, severely narrowing the urethral lumen and preventing the entry of the scope into the proximal urethra. A biopsy sample was obtained and underwent cytological examination. The results were suggestive of urothelial hyperplasia.

Based on the dog's history, the results of the clinical examination and diagnostic imaging a partial urethral obstruction, caused by stent‐related tissue hyperplasia, associated with overflow urinary incontinence secondary to chronic bladder overdistention and subsequent detrusor atony, was suspected. Treatment of the urinary tract infection was started with marbofloxacin (3 mg/kg orally once daily) 24 h before surgery. The urine scald was treated with a topical ointment 1% chlorhexidine and vaseline‐based twice daily.

**FIGURE 2:**
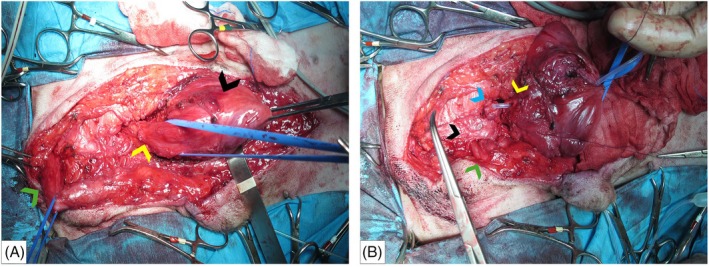
Intraoperative view after the caudal celiotomy. (A) The urinary bladder (black arrowhead), the proximal urethra (yellow arrowhead), and the root of the penis (green arrowhead) are isolated. Note the abundant fibrous scar tissue at the level of the pubic region which developed after the previous pubectomy (the right side of the image shows the cranial view). (B) The mobilized penis and the related urethra (green arrowhead) are tunnelized into the abdominal cavity, passing through the abdominal wall muscles (black arrowhead) at the level of the inguinal region, shown on the left side. The proximal end of the penile urethra (blue arrowhead) is then located in proximity of the vesicourethral junction (yellow arrowhead) allowing the subsequent end‐to‐end urethral anastomosis. Note the indwelling 10F urethral Foley catheter advanced retrogradely into the urinary bladder (right side of the image shows the cranial view).

Transabdominal urethral anastomosis, after abdominal wall passage of the penis, was planned as a surgical alternative to resolve the urethral obstruction. The dog was anesthetized and the abdomen, inguinal region and perineum were aseptically prepared for surgery. Cephazolin (22 mg/kg IV) was administered preoperatively and repeated every 90 min during surgery. The patient was positioned in dorsal recumbency and a ventral midline caudal abdominal celiotomy was performed. The incision was extended caudally to the ventral perineum, at the level of the root of the penis. The region of the pelvic symphysis was exposed by elevation of the adductor, gracilis and parts of the external obturator muscles; access to the pelvic cavity was obtained by blunt and sharp dissection through the fibrous tissue which had developed at the level of the previous pubectomy. The urinary bladder wall was thickened and atonic and multiple adhesions with the parietal peritoneum were noted. The proximal pelvic urethra appeared dilated and numerous adhesions with the adjacent connective tissue were also observed. The adhesions were resolved using blunt dissection and bipolar electrocauterization, taking care to avoid the ureters and the uretero‐vesical junctions; the urinary bladder and the proximal urethra were then isolated, and the vesico‐urethral junction identified (Figure 2A). A caudal ventral cystotomy was performed and an antegrade urethral catheterization with a 5F urethral catheter was attempted; however, it failed to pass through the site of the obstruction. Simultaneously, a 5F urethral catheter was advanced into the urethra in retrograde fashion until the site of the obstruction was encountered. The obstructed urethral tract extended from 15 mm caudal to the trigone up to the ischiatic curvature, corresponding to the entire length of the urethral stent. This tract of the urethra was double ligated and incised 5 mm proximally and 20 mm distally to the stent; however, it was not excised due to adherences with the ventral rectal wall. A full thickness urethral biopsy was collected and submitted for histologic examination. The root of the penis was then isolated and both the ischiocavernosus and ischiourethralis muscles were transected near their ischiatic origin. The dorsal penile vessels were bluntly dissected from the underlying penile tissue and preserved. The retractor muscle of the penis was transected, and the remaining penile attachments freed from the ischium. The penis was released by transecting the bulbospongius muscle and the proximal part of the penile body, dissecting the surrounding soft tissue cranially to the level of the os penis. Two partial‐thickness stay sutures, spaced 45° around the dorsal circumference of the caudal end of the released penis, were placed with 3–0 monofilament Glycomer 631 (Biosyn, Covidien USA, Minneapolis, Minnesota). Carmalt forceps were then advanced from the abdominal cavity into the inguinal area, through the abdominal wall muscle creating a tunnel 2 cm lateral to the linea alba on the left side; both the stay‐sutures were grasped. The penis was mobilized, passed through the abdominal wall tunnel into the abdomen, to the level of the remaining proximal urethra, withdrawing the Carmalt forceps. Attention was paid to make the passage through the abdominal wall of an adequate size to avoid compression of the penile vascularization. A 10F indwelling Foley catheter was then advanced in retrograde fashion into the urinary bladder through the penile urethra. Without dissecting the penile urethra from the cavernous and spongiosum tissue, an intra‐abdominal end‐to‐end apposition anastomosis, between the vesico‐urethral junction and the penile urethra, was performed with 4–0 Glycomer 631 (Biosyn, Covidien USA) in a full‐thickness simple interrupted pattern (Figure 2B). Suture was performed with care to avoid any tension on the anastomosis. A 12F cystostomy catheter was subsequently placed as a urinary diversion system; the multiple cystic calculi had been removed by the previous cystotomy and submitted for stone analysis. Three full‐thickness bladder biopsy samples were also obtained for histologic examination and aerobic and anaerobic microbial culturing, as was one crushed stone. The cystotomy was closed with 3–0 Glycomer 631 (Biosyn, Covidien USA) in a simple interrupted pattern, the surgical site was irrigated with a warm sterile saline solution, and the celiotomy was closed routinely.

The dog recovered uneventfully from the anesthesia and received fentanyl (3 μg/kg/h IV constant rate infusion) along with marbofloxacin (3 mg/kg IV, every 24 h) and Ringer's Lactate solution (2 mL/kg/h) postoperatively. Urine output was monitored during hospitalization. Then, 24 h postoperatively, the patient was transitioned to methadone (0.2 mg/kg IM pro re nata (PNR)), by the attending clinician on the basis of Glasgow pain scoring system evaluation.

Abdominal ultrasound, performed at 5 days postoperatively, revealed mild soft, tissue thickening at the level of the urethral anastomosis without any evidence of free abdominal fluid. The results of the histologic examination of both the urethral and the bladder biopsy samples obtained during surgery were consistent with severe fibrosis, pyogranulomatous inflammation and urothelial hyperplasia. The stone analysis revealed that the uroliths were 100% struvite. Bacterial culture yielded the growth of *Staphylococcus pseudointermedius*, susceptible to marbofloxacin from both the urolith and bladder samples. Antimicrobial therapy was maintained, as described above.

## 3 RESULTS

**FIGURE 3:**
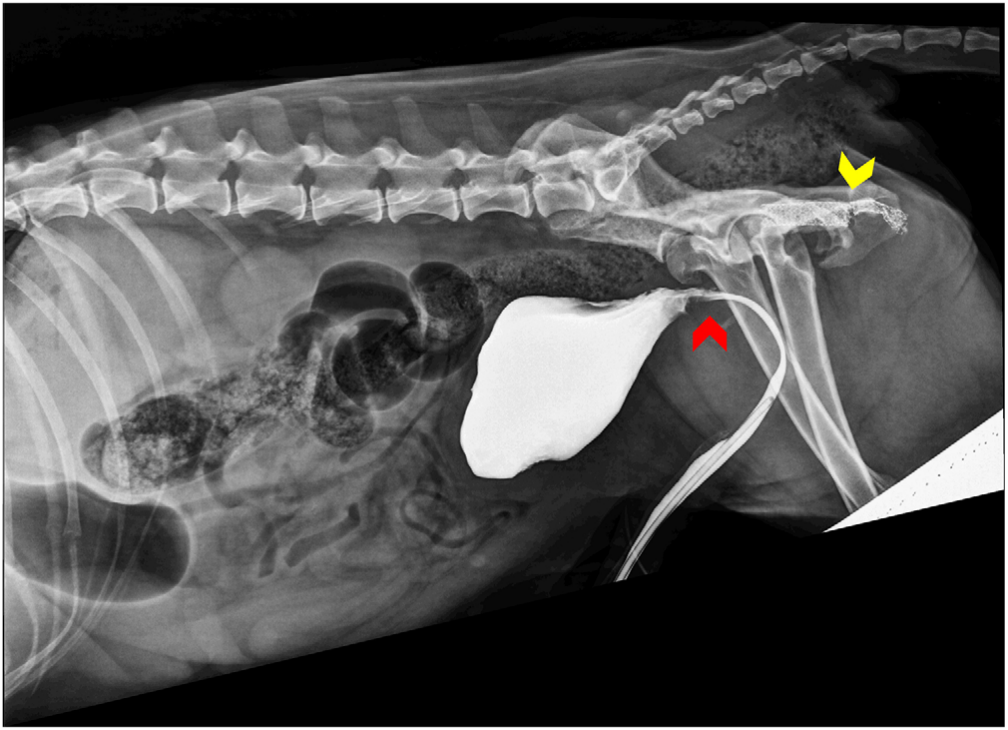
Postoperative abdominal radiographs. A contrast cystourethrogram 10 days post‐surgery, obtained by withdrawing the indwelling urethral Foley catheter distally. Note the new course of the urethra and the absence of contrast's leakage from the urethral anastomosis (red arrowhead). At this level, the margins of the urethra appear moderately irregular. The fractured urethral self‐expanding metallic stent (yellow arrowhead) is still visible in its original location.

Ten days postoperatively, the indwelling 10F urethral catheter was removed, and a contrast retrograde urethro‐cystography was performed. The urethral anastomosis appeared to be healed, and no contrast leakage was observed (Figure 3). Subsequently, the dog postured to urinate normally but was unable to produce a urine stream, and mild urinary incontinence had also been noted during recumbency (7 of 10 based on the continence score proposed by Rose et al.).^7^ The urinary bladder was easily emptied by manual expression and bethanechol (5 mg/kg orally, twice daily) was introduced to enhance stimulation of the detrusor contraction, because bladder atony was suspected.

The dog was discharged 15 days after surgery with the cystostomy catheter left in place with marbofloxacin and bethanechol being continued. The owners were instructed to manually express the urinary bladder at least three times a day and to use the cystostomy tube to completely empty the bladder once a day. A recheck examination was performed at 4 weeks postoperatively and the clinical condition appeared to be good, no signs of urine dermatitis were noted and the dog was reported to be still mildly incontinent (7 of 10 based on the continence score proposed by Rose et al.).^7^ The owners were able to completely empty the urinary bladder by manual expression and the cystostomy catheter was removed. No bacterial growth was yielded from the urine collected at that time and the antimicrobial treatment was discontinued.

At 6 month follow‐up the dog remained mildly incontinent and unable to voluntarily urinate; however, the urinary bladder was easily and fully emptied by manual expression. Moreover, the owners discontinued bethanechol contrary to the recommendations of the clinicians after 3 months of treatment. The urine stream, obtained by manual bladder expression, was considered subjectively normal and no clinical or laboratory signs of recurring UTIs were noted. The abdominal ultrasonography confirmed complete voiding of the bladder contents.

Nine months postoperatively the dog was stable regarding urological signs and micturition; however, it had multiple episodes of cluster‐seizures and generalized epilepsy, requiring a dose escalation of antiepileptic drugs. After the occurrence of refractory status epilepticus, the owners refused any other clinical investigation and elected euthanasia.

## 4 DISCUSSION

In this case report, a modified surgical technique of transabdominal urethral anastomosis after abdominal wall passage of the penis in a dog was described. This novel technique was useful in restoring urethral patency and avoiding urethrostomy. This strategy succeeded despite extensive abnormalities in the pelvic urethra and a very short pelvic urethra.

Intraluminal urethral stenting has been demonstrated to be a minimally‐invasive and effective treatment for the relief of benign urethral obstruction; however, few data exist regarding long‐term complications in dogs.^8^, ^9^, ^10^ In humans, long term complications associated with the placement of uncovered metallic stents for benign urethral strictures are as high as 55%, the majority of the complications stemming from stent obstruction secondary to tissue hyperplasia and in‐growth through the stent interstices.^6^ Tissue ingrowth can occur when there is a gap between the stent and the wall of the organ' it is a complication commonly described in dogs having tracheal stents.^11^ Medical treatment with glucocorticoids is normally advised; it can be effective in reducing tissue hyperplasia in 50% of the cases.^11^ This approach is not suggested in dogs having urethral stents, probably because it is utilized for the most part employed in cases of malignant disease or because local immunosuppression, derived by long‐term corticosteroids administration, could promote ascending UTIs.^12^

Treating a urethral obstruction secondary to hyperplastic overgrowth following stent placement was reported using a second covered stent placed within the previously placed stent.^9^, ^13^ However this option was not feasible in the case reported herein for two main reasons. First, it was impossible to advance the guide wire through the urethra at the level of the stent, indicating a severe obstruction. Second, the fragment of the fractured stent displaced along the urethra could have eventually compromised the correct deployment of the new stent. Although in human beings it is sometimes possible to remove the stent piecemeal endoscopically wire by wire, the en bloc removal of the scarred urethra, together with the entrapped stent, and a subsequent urethroplasty or urethrostomy, are frequently required.^6^

Prepubic urethrostomy is a viable option in dogs for restoring urethral patency when the proximal urethra is too short, preventing urethral resection and anastomosis, or a scrotal urethrostomy.^1^ This would have been a viable option in the dog discussed in this report if the intra‐abdominal anastomosis had not been possible. As first choice, prepubic urethrostomy was considered suboptimal due to the ventral urine dermatitis and the significant risk of postoperative complications, such as urinary tract obstruction, urine scalding or recurrent UTIs,^1^ which could have impacted the dog's quality of life. Giansetto et al. recently described a modified preputial urethrostomy in dogs, with the intention of overcoming these complications; however, they reported a difficult retrograde urinary catheterization after surgery as a specific disadvantage of their technique.^3^ This potential complication would have been a significant disadvantage in this dog which had chronic detrusor atony and might have required repeated catheterization in the future.

As an alternative, a technique of penile diversion into the abdominal cavity through the celiotomy site has been described by Bacon et al., who performed a uretero‐urethral anastomosis to an internalized penile urethra after a total cystectomy and prostatectomy in two male dogs.^14^ In that report, the penile urethra was just folded across the laparotomy at the level of the pubic brim, and the abdominal wall was closed normally taking care to not compress the penis at the caudal end of the closure.^14^ Although the previous pubectomy facilitated the penile mobilization, the absence of the pubis and the abundance of scar tissue in the dog reported herein, persuaded the authors to avoid the interposition of the penis through the celiotomy site, and the transabdominal wall passage was chosen as a viable alternative to internalize the penis into the abdominal cavity, allowing urethral anastomosis.

The scar tissue subsequent to the previous surgery also prevented the use of the inguinal ring as a natural passage for the penile urethra as suggested by Minier et al.^4^ In that paper, the authors described a case of periurethral liposarcoma in a dog, affecting the membranous part of the urethra, which was treated by an extrapelvic end‐to‐end anastomosis after inguinal tunnelization of the cranial pelvic urethra. Similarities can be found in the case reported herein including the site of the urethral obstruction and the need to bypass a long part of the pelvic urethra. However, the previous prostatectomy and the shortness of the remaining healthy pelvic urethra in the dog described herein, induced the authors to mobilize the penis into the abdominal cavity and realize a transabdominal urethral anastomosis.^4^ The passage of the penis through the abdominal wall could have increased the risk of urethral obstruction due to the muscle compression; however, the stiffness of the penis, given primarily by the os penis, may have prevented this long‐term complication in the case herein described.

To allow penile mobilization without compromising the vascular supply, the dorsal blood vessels were bluntly dissected and separated from the underlying penile tissue, preserving their integrity. Damage to the dorsal penile vessels could have determined transient ischemia of the penis, with a negative impact on the success of the surgery.^15^ However, the primary blood supply to the penis is from three branches of the artery of the penis, which is a direct continuation of the internal pudendal artery. These three branches, the artery of the bulb, deep artery of the penis and dorsal artery of the penis, anastomose with one other, ensuring adequate blood supply, even when one of these vessels is temporarily damaged.^16^

In the present case, both cystostomy and indwelling urethral catheters were chosen as urinary diversion techniques for postoperative management in order to avoid urine contact with the anastomotic site. Although the use of these catheters during urethral healing is controversial, they seem to decrease the risk of urethral stricture.^1^, ^17^ Given the bladder atony, the cystostomy catheter was maintained for 28 days in the dog discussed here, with the goal of improving urinary drainage and detrusor muscle recovery. Detrusor atony, secondary to the chronic urinary outflow obstruction, was suspected in this dog owing to the duration of the clinical signs. Relief of the obstruction and maintenance of a small bladder volume for up to 2 weeks are suggested as the treatment of choice to permit the return of coordinated detrusor function.^18^ Bethanechol is also advised, after relieving the obstruction, to enhance the stimulation of the detrusor contraction only if the pelvic nerves are intact.^18^ Unfortunately, despite all the treatment being pursued, the dog in this report did not regain the ability to urinate spontaneously. This could have been related to the duration of the bladder overdistention which may have caused irreversible damage to the tight junctions of the detrusor' muscle fibers, or related to pelvic nerve damage.^18^ Although the dog remained unable to voluntarily urinate, the surgery allowed the resolution of the urethral obstruction and easy voiding of the urinary bladder by manual expression performed by the owner, thus avoiding the need for a lifelong cystostomy tube.

Urinary incontinence improved postoperatively; however, as expected, it did not completely resolve. At the time of the presentation, the urinary incontinence was attributed to the outflow obstruction and was classified as severe due to the continuous dribbling of urine. After surgery, urinary incontinence was still evident; however, only occurred when the dog was recumbent. Furthermore, the dog's incontinence had a longer duration, starting when he was 1 year of age and lasting 6 years after the prostatectomy had been performed as a result of severe trauma. Urethral injuries and possible damage to the bladder innervation could have occurred during a previous traumatic event or prostatectomy. Urinary incontinence should also be considered to be a postoperative complication of the technique used here as a long portion of the urethra was resected. Unfortunately, the authors were unable to evaluate this aspect in the present case due to the previous occurrence of urinary incontinence prior to presentation.

En bloc removal of the scarred urethra and the entrapped fractured stent was not perused in this dog due to the concern of creating iatrogenic damage to the ventral rectal wall due to the presence of the adhesions. This choice could have led to the development of abscess or fistula formation, because the urethral segment containing the infected stent was double ligated and was not removed. This complication was not observed in this dog; however, it might have been influenced by the short‐term follow‐up.

In conclusion, the present report suggests that an end‐to‐end intra‐abdominal urethral anastomosis after abdominal wall passage of the penis can be considered to be a valuable surgical option for dogs in which the pelvic tract of the urethra appears severely damaged or must be excised. This technique can be indicated in the case of extensive trauma, in the case of stent‐related urethral obstruction, or in the case of periurethral neoplasia, in the pursuit of a curative intent. Further studies with larger case numbers are necessary to fully explore the outcome and the complications of this urologic surgical technique in dogs.

## AUTHOR CONTRIBUTIONS

Foglia A, DVM, PhD: Conceived the surgical technique, performed the surgery, drafted and revised the manuscript. Cola V, DVM, PhD: Collected the data, manuscript revision and editing. Del Magno S, DVM, PhD, DECVS (Small Animal): Collected data, manuscript revision and editing. Dondi F, DVM, PhD: Involved in the clinical management of the dog as attending clinician and manuscript revision. Troia R, DVM, PhD, DECVECC: Involved in the clinical management of the dog as attending clinician and manuscript revision. Zanardi S, DVM, GpCert(SASTS): collected data and manuscript revision. Cinti F, DVM, PhD, CpCert (SASTS), DECVS (Small Animal), MRCVS: Manuscript revision. Pisoni L DVM, PhD: Conceived the surgical technique, performed the surgery and revised the manuscript. All the authors provided a critical review of the manuscript and endorse the final version.

## CONFLICT OF INTEREST STATEMENT

The authors did not receive any grant or financial support and declare no conflicts of interest.
